# Postoperative nomogram to predict the probability of metastasis in Enneking stage IIB extremity osteosarcoma

**DOI:** 10.1186/1471-2407-14-666

**Published:** 2014-09-12

**Authors:** Seung Hyun Kim, Kyoo-Ho Shin, Ha Yan Kim, Yong Jin Cho, Jae Kyoung Noh, Jin-Suck Suh, Woo-Ick Yang

**Affiliations:** Department of Orthopaedic Surgery, Yonsei University College of Medicine, 50 Yonsei-Ro, Seodaemun-Gu, Seoul, Korea; Biostatistics Collaboration Unit, Yonsei University College of Medicine, Seoul, Korea; Cancer Center, Yonsei University College of Medicine, Seoul, Korea; Department of Radiology and Research Institute of Radiological Science, Yonsei University College of Medicine, Seoul, Korea; Department of Pathology, Yonsei University College of Medicine, Seoul, Korea

**Keywords:** Osteosarcoma, Metastasis, Nomogram, Dichotomous outcomes

## Abstract

**Background:**

Metastasis is the most crucial prognostic factor in osteosarcoma. The goal of this study was to develop a new nomogram to predict the probability of metastasis in Enneking stage IIB extremity osteosarcoma after neoadjuvant chemotherapy and limb salvage surgery.

**Methods:**

We examined medical records of 91 patients who had undergone surgery between March 1994 and March 2007. A nomogram was developed using multivariate logistic regression. The nomogram was validated internally by bootstrapping-method (200 repetitions) and externally in independent validation set (n = 34). A Youden-derived cutoff value was assigned to the nomogram to predict dichotomous outcomes for metastasis.

**Results:**

The nomogram was built from four predictors of tumor site, serum alkaline phosphatase, intracapsular extension, and Huvos grade, and an additional clause that the cutoff value should be added to the total points in the cases of incomplete surgical resection. *P*-value of Hosmer and Lemshow Goodness-of-fit test of this model was 0.649. Area under receiver operating curve values of 0.83 (95% confidence interval [CI], 0.75 to 0.92) in the training set and 0.80 (95% CI, 0.63 to 0.96) in the validation set were obtained. The accuracy of dichotomous outcomes was 79.1% (95% CI, 0.69 to 0.86) and 82.4% (95% CI, 0.63 to 0.92) in the training and validation sets.

**Conclusions:**

We have developed a new high-performance nomogram to predict the probability of metastasis in Enneking stage IIB extremity osteosarcoma after limb salvage surgery.

## Background

Although osteosarcoma is a rare disease, it is the most common primary malignant bone tumor. Prior to 1970, the oncologic outcomes of osteosarcoma were extremely poor with only a 10-20% overall survival rate despite aggressive surgery. The overall survival rates of osteosarcoma have dramatically increased to approximately 65-75% with the establishment of multidisciplinary treatments [1].

The Enneking staging system and American Joint Committee on Cancer (AJCC) are used to classify osteosarcoma according to prognosis primarily based on histologic grade and metastasis at diagnosis [2, 3]. In addition to the factors used for clinical staging, many other clinical factors have been reported to be prognostic factors for osteosarcoma such as age, [4] tumor location, [5–7] serum markers such as alkaline phosphatase (ALP) [8] and lactate dehydrogenase (LDH), [9] pathologic fracture, [10] histologic type, [11] and histologic response to neoadjuvant chemotherapy [12]. Molecular markers of prognosis in osteosarcoma have also been reported including ezrin, chemokine receptor 4, and P-glycoprotein [13]. Because no single factor can accurately predict prognosis, statistical prediction models to integrate the cumulative effects of individual prognostic factors are required for more precise prognosis predictions. Nomograms have been proposed as a new and alternative tool to traditional staging systems for predicting prognosis in a variety of cancers [14]. A few nomograms have been reported for soft tissue sarcoma [15, 16] and osteosarcoma [17].

Although multidisciplinary approach has dramatically improved survival in osteosarcoma, the presence of metastasis makes this a challenging disease to cure, for survival rates of osteosarcoma with metastasis are of approximately 20% [18]. On the other hand, osteosarcoma without metastasis can be cured and most osteosarcoma patients without metastasis live a long and healthy life. Therefore, the accurate prediction of an individual patient’s probability of metastasis is important. The purpose of this study was to develop a new nomogram to predict the probability of metastasis in Enneking stage IIB extremity osteosarcomas, which rank the majority of osteosarcoma cases.

## Methods

### Patients

**Figure 1:**
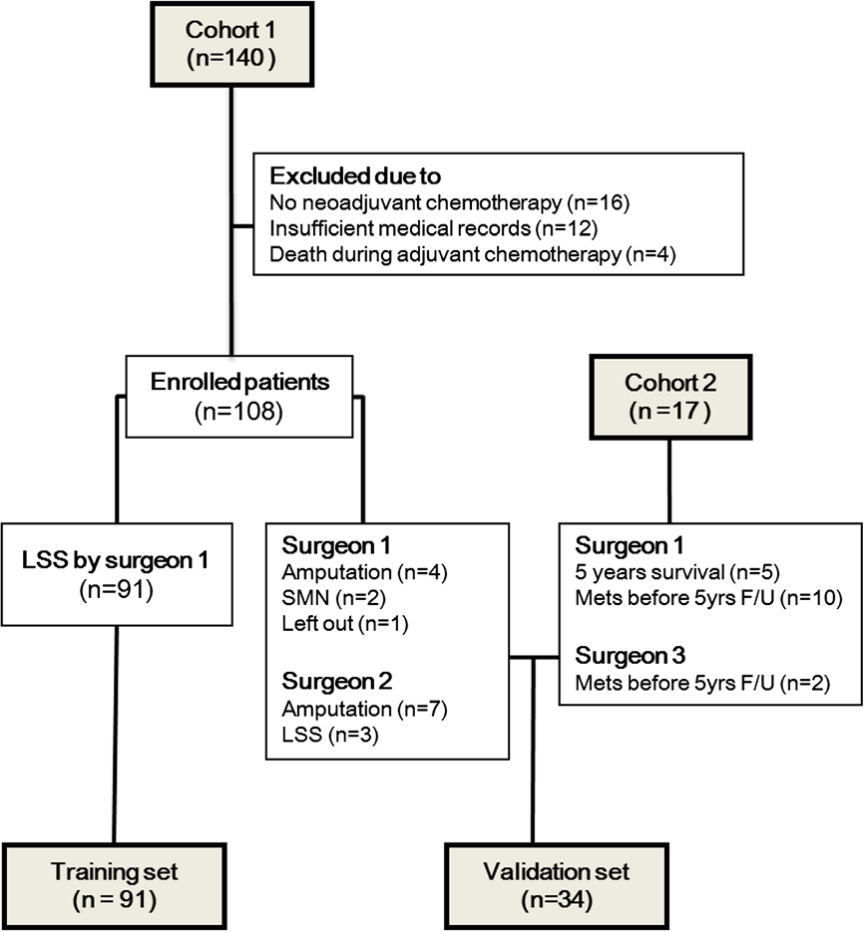
**Diagram for populations of training and validation set.** Cohort 1 included the patients with Enneking IIB osteosarcoma who had have surgery between March 1994 and March 2007 at Severance Hospital (Seoul, Korea) and Cohort 2 included the patients with Enneking IIB osteosarcoma who had have surgery between April 2007 and March 2011 at the same institute. LSS, limb salvage surgery, SMN, secondary malignant neoplasm, mets, metastasis, F/U, follow up.

We searched and retrospectively reviewed the medical records of Enneking stage IIB extremity osteosarcoma patients who had undergone surgery between March 1994 and March 2007 (cohort 1) at Severance Hospital (Seoul, Korea). This study was done under Severance Hospital Institutional Review Board-approved protocol. We restricted the inclusion criteria for the training set to the patients who had undergone standard therapy (neoadjuvant chemotherapy, definitive surgery, and adjuvant chemotherapy) and limb salvage surgery that was performed by the same surgeon. Of the 140 patients identified, 108 patients were enrolled in the study. Of the 108 patients, 91 and 17 patients were included in the training and validation sets, respectively, according to the inclusion criteria. An additional 17 patients who had undergone surgery between April 2007 and July 2011 (cohort 2) at Severance Hospital were included in the validation set (Figure 1). The clinical characteristics of the training and validation sets are listed in Table 1. The overall 5-year survival rate of the training set was 70.3%. The proportions of patients with metastasis in the training and validation sets were 37.4% and 50%, respectively. Because the follow-up period of cohort 2 (with the longest follow-up period of 7 years) was much shorter than that of cohort 1 (with the longest follow-up period of 19 years), fewer patients with 5-year continuously disease free (CDF) status after definitive surgery and 5-year no evidence of disease (NED) status after last metastasectomy were enrolled in cohort 2 than cohort 1, which led to quite a difference in the proportions of patients with metastasis.

**Table 1:** **Clinical characteristics of training and validation sets**

Variables		Training (***N***= 91)		Validation (***N***= 34)	
		***N***	%	***N***	%
Survival	5 years survivor (CDF from definitive surgery)	58	63.7	16	47.1
	5 years survivor (NED after metastasectomy)	6	6.6	2	5.9
	DOD	27	29.7	7	20.6
	NED (after metastasectomy) < 5 years	0	0.0	6	17.6
	AWD	0	0.0	2	5.9
	DOC	0	0.0	1	2.9
Metastasis	Positive	34	37.4	17	50.0
	Free	57	62.6	17	50.0
Sex	Male	50	54.9	19	55.9
	Female	41	45.1	15	44.1
Age	≤ 14 yrs	36	39.6	17	50.0
	> 15 yrs	55	60.4	17	50.0
Tumor site	Distal femur	46	50.5	12	35.3
	Proximal tibia	17	18.7	7	20.6
	Proximal humerus	14	15.4	5	14.7
	Others	14	15.4	10	29.4
Tumor size	≥ 8cm	62	68.1	20	58.8
	< 8cm	29	31.9	14	41.2
Pathologic fx at diagnosis	Yes	5	5.5	2	5.9
	No	86	94.5	32	94.1
Skip lesion	Yes	3	3.3	2	5.9
	No	88	96.7	32	94.1
Intracapsular extension	Yes	20	22.0	10	29.4
	No	71	78.0	24	70.6
ALP	Elevation	56	61.5	11	32.4
	Normal	35	38.5	23	67.6
LDH	Elevation	9	9.9	5	14.7
	Normal	53	58.2	16	47.1
	NA	29	31.9	13	38.2
Histologic type	Osteoblastic	62	68.1	17	50.0
	Chondroblastic	11	12.1	3	8.8
	Fibroblastic	4	4.4	1	2.9
	Mixed	12	13.2	5	14.7
	Others	2	2.2	3	8.8
	NA	0	0.0	5	14.7
Huvos grade	III and IV	65	71.4	21	61.8
	I and II	26	28.6	13	38.2
Operation Type	Limb salvage surgery	91	100.0	23	67.6
	Amputation	0	0.0	11	32.4
Surgeon factor	Surgeon 1	91	100.0	22	64.7
	Surgeon 2	0	0.0	10	29.4
	Surgeon 3	0	0.0	2	5.9
Surgical resection	R0	85	93.4	33	97.1
	R1	6	6.6	1	2.9

Abbreviation: CDF, continuously disease free, DOD, died of disease, NED, no evidence of disease, AWD, alive with metastatic disease, DOC, died of other cause, fx, fracture, ALP, alkaline phosphatase, LDH, lactate dehydrogenase, NA, not available.

No patients received radiation therapy at the primary tumor site. Only seven patients in the training set received palliative radiation therapy on the metastatic lesions. All the patients received neoadjuvant chemotherapy. Sixty-five patients were treated with doublet of intra-arterial cisplatin (DDP) and doxorubicin (ADR), fifty patients were treated with triplet intra-arterial DDP, ADR, and ifosafamide (Ifos). Ten patients were treated with other regimens: five patients with ADR and intravenous DDP; four patients with ADR, intravenous DDP, and methotrexate (MTX); and one patient with VP-16, Ifos, and MTX. Huvos grade, disease-free survival, and overall survival were not significantly different between doublet and triplet regimens in our cohorts [19].

### Developing the nomogram

We identified candidate predictors of metastasis using the χ^2^ test and performed multivariate analysis of a variety of suggested candidates (Table 2). Among these candidates, we chose the parameters for a nomogram that were statistically significant and developed a weighted nomogram. The association between these parameters and metastasis was evaluated using multivariate logistic regression analysis. A nomogram was developed on the basis of the multivariate logistic regression model using tumor site, ALP at diagnosis, intracapsular extension, and Huvos grade. The goodness-of-fit of the nomogram was calculated using the Hosmer-Lemeshow test.

**Table 2:** **χ2 tests for identification of prognostic factors for metastasis**

Candidate		Metastasis positive (%)	Metastasis free (%)	***P***
Sex	Male	21 (42.0)	29 (58.0)	0.31
	Female	13 (31.7)	28 (68.3)	
Age	≤ 14 years	12 (33.3)	24 (66.7)	0.52
	> 15 years	22 (40.0)	33 (60.0)	
Tumor site	Distal femur/Proximal tibia/Proximal humerus (Not exceeding the isthmus)	12 (21.1)	45 (78.9)	<0.001
	Others	22 (67.4)	12 (35.3)	
Laterality	Left	13 (32.5)	27 (67.5)	0.40
	Right	21 (41.2)	30 (58.8)	
Tumor size	≥ 8 cm	25 (40.3)	37 (59.7)	0.39
	< 8 cm	9 (31.0)	20 (69.0)	
Pathologic fracture at diagnosis	Yes	3 (60.0)	2 (40.0)	0.36^*^
	No	31 (36.0)	55 (64.0)	
Skip lesion	Yes	2 (66.7)	1 (33.3)	0.55^*^
	No	32 (36.4)	56 (63.6)	
Intracapsular extension	Yes	13 (65.0)	7 (35.0)	0.004
	No	21 (29.6)	50 (70.4)	
ALP at diagnosis	Elevated	26 (46.4)	30 (53.6)	0.02
	Normal	8 (22.9)	27 (77.1)	
LDH at diagnosis	Elevated	5 (55.6)	4 (44.4)	0.12^*^
	Normal	14 (26.4)	39 (73.6)	
Histologic type	Osteoblastic/chondroblastic/fibroblastic	19 (29.2)	46 (70.8)	0.02
	Others (mixed and nonconventional type)	9 (60.0)	6 (40.0)	
Limb salvage surgery type	Without Pasteurization	28 (36.4)	49 (63.6)	0.64
	With Pasteurization	6 (42.9)	8 (57.1)	
Huvos grade	I & II	15 (57.7)	11 (42.3)	0.01
	III & IV	19 (29.2)	46 (70.8)	
Surgical resection	Complete	28 (32.9)	57 (67.1)	0.002^*^
	Incomplete	6 (100.0)	0 (0.0)	

*Abbreviation*: *ALP* alkaline phosphatase, *LDH* lactate dehydrogenase.

^*^Calculated using Fisher’s extract test.

### Definitions of the parameters for each predictor in the nomogram

The parameters of all predictors were divided into two prognosis groups, good or poor.

#### Tumor site

Tumors located along the distal femur, proximal tibia, and proximal humerus were regarded as the good prognosis group and those at other locations were regarded as the poor prognosis group. In addition, tumors along the distal femur, proximal tibia, and proximal humerus with a longitudinal size that it exceeded the isthmus of the affected bone (more than half the entire length of the affected bone) were categorized in the poor prognostic group.

#### Intracapsular extension

Intracapsular extension was regarded as the poor prognosis group. Intracapsular extension of the tumor was defined not only as direct penetration of the articular cartilage but also as the involvement of intracapsular and extrasynovial structures. Diagnosis of intracapsular extension by MRI, whether positive or negative, was confirmed by gross pathology.

#### Serum ALP levels at diagnosis

Normal level of alkaline phosphatase (ALP) was regarded as the good prognosis group. The serum ALP levels were measured in international units (IU), and the activity of ALP was estimated by the p-nitrophenyl phosphate method. ALP ranges of 60.0-300.0 IU/L for patients ≤14 years and 38.0-115.5 IU/L for patients > 15 years were considered normal.

#### Response to neoadjuvant chemotherapy

Responses to neoadjuvant chemotherapy were graded on the basis of the amount of tumor necrosis in the resected specimen. More than 90% tumor necrosis was regarded as a good response; a cut-off of 90% tumor necrosis is usually used to distinguish good and poor responders. Good response was categorized in the good prognosis group.

#### Surgical resection

Surgical resection was assessed by resection margin from pathology not surgical margin. Free of tumor (R0) was defined as complete surgical resection, while positive margins microscopically (R1) and macroscopically (R2) were defined as incomplete surgical resection. Complete surgical resection was regarded as good prognosis group.

### Statistical analysis

The performance of our nomogram was evaluated internally and externally for discrimination and calibration. Discrimination was evaluated by the area under receiver operating characteristic curve (AUC) for both the training set (*N* = 91) and the external validation set (*N* = 34). A 95% confidence interval (CI) was calculated for each AUC. Calibration plots were obtained from bootstrapping (200 repetitions) of the training and validation sets.

To improve the clinical practicality of the nomogram, we assigned a cutoff value, derived from the Youden index, to the nomogram to allow for the prediction of dichotomous outcomes for metastasis. Nomogram performance in predicting dichotomous outcomes was also evaluated in the training and validation sets by two-way contingency table analysis. A 95% CI was calculated for each indicator.

All statistical analysis were performed using SPSS (version 20.0, SPSS, Inc., Chicago, IL, USA), SAS (version 9.2,SAS Institute Inc., Cary, NC, USA), and R (version 2.9.1,The R Foundation for Statistical Computing, Vienna, Austria). All *P* values were two-tailed, and a *P* value < 0 .05 was considered significant.

## Results

### Nomogram development and validation

**Figure 2:**
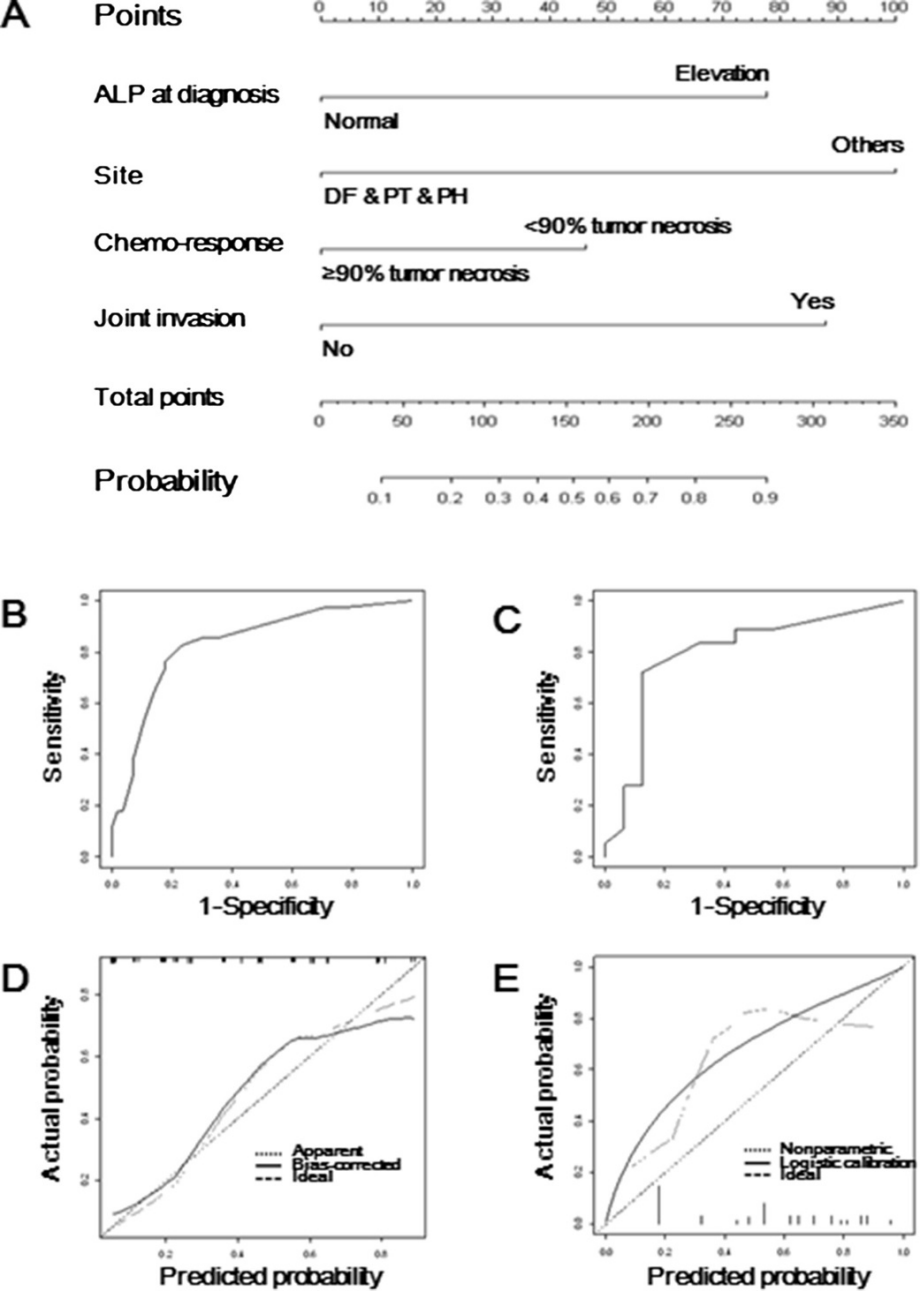
**Nomogram to predict probability of metastasis and validations. (A)** The postoperative monogram **(B)** ROC curve for the training set of 91 patients **(C)** ROC curve for the validation set of 34 patients **(D)** calibration plot for the training set **(E)** calibration plot for the validation set. ROC curve, receiver operating characteristic curve.

Six factors of tumor site, ALP level at diagnosis, intracapsular extension, Huvos grade, histologic type, and surgical resection were identified as prognostic factors for metastasis (Table 2). The odds ratios for metastasis were calculated for these and are shown in Table 3. The odds ratio of surgical resection was beyond compute, because all the cases with incomplete surgical resection had undergone metastasis. Huvos grade and histologic type were strongly correlated and confounded the multivariate analysis. Therefore, surgical resection and histologic type were excluded from the prediction model. On the basis of multivariate logistic regression analysis, we built a nomogram using tumor site, ALP level at diagnosis, intracapsular extension, and Huvos grade as the predictors (Figure 2A). The *P* value of the Hosmer-Lemeshow test for the prediction model was 0.65, which indicated the good statistical fit of the model.AUC values of 0.83 (95% CI, 0.75 to 0.92) and 0.80 (95% CI, 0.63 to 0.96) were obtained in the training and validation sets, respectively (Figure 2B and C). The calibration plot for the training and validation sets is shown in Figure 2D and E, respectively. The bootstrap-corrected AUC was 0.81. There was no significant difference among the three AUC values, which suggested that the discrimination of the nomogram could be reproducible in other populations. The calibration plots showed that the nomogram predicted probabilities were slightly lower than the actual probabilities.

**Table 3:** **RR and OR of prognostic factors for metastasis**

	RR (95% CI)	Univariate analysis		Multivariate analysis ^*^	
		OR (95% CI)	***P***	OR (95% CI)	***P***
Constant				0.00	0.000
Tumor site	3.07 (1.75 to 5.38)	6.88 (2.66 to 17.76)	0.000	6.49 (2.13 to 19.78)	0.001
ALP at diagnosis	2.03 (1.04 to 3.97)	2.93 (1.13 to 7.55)	0.03	4.27 (1.34 to 13.64)	0.01
Intracapsular extension	2.20 (1.36 to 3.56)	4.42 (1.55 to 12.65)	0.006	5.19 (1.47 to 18.27)	0.01
Huvos grade	1.97 (1.20 to 3.26)	3.30 (1.29 to 8.49)	0.01	2.37 (0.73 to 7.67)	0.15
Histologic type	2.05 (1.17 to 3.59)	3.74 (1.14 to 12.34)	0.03		
Surgical resection	3.04 (2.24 to 4.11)	NA	NA		

*Abbreviation*: *RR* relative risk, *OR* odds ratio, *CI* confidential interval, *ALP* alkaline phosphatase, *NA* not applicable. ^*^ *P*-value of Hosmer and Lemshow Goodness-of-fit test is 0.649.

### Cutoff value for dichotomous outcomes

Nomograms show the probability of metastasis as a percentage; however, dichotomous outcomes for metastasis are likely to be a user friendly option in practice. Therefore, we assigned a Youden-derived cutoff value to the nomogram. The cutoff value was a total of 123 points, which was equal to a predicted probability of 0.36. The combined score of the two poor prognosis parameters with the lowest scores was more than the cutoff value. Therefore, the dichotomous decision for metastasis is positive whenever any two of the four predictors are classified as poor group.

The relative risk comparisons for the predictors showed that surgical resection was a very strong prognostic factor (Table 3). However, surgical resection had to be excluded from the nomogram for statistical reasons because all six cases with an incomplete surgical margin showed metastasis: Odds ratios are calculated as the probability of metastasis/(1-the probability of metastasis). Therefore, for these cases, the probability of metastasis would be 100%, and the odds ratio would not be mathematically calculable, as the denominator would be zero. To overcome this problem, we imposed an additional clause on the nomogram that the cutoff value should be added to the total points in the cases of incomplete surgical resection. Consequently, all the cases with incomplete resection margin were always metastasis positive in the dichotomous outcomes.

The performance of the nomogram in predicting dichotomous outcomes for metastasis was validated by two-way contingency table analysis (Table 4). The accuracy of the nomogram in predicting dichotomous outcomes for metastasis was 79.1% (95% CI, 0.69 to 0.86) in the training set and 82.4% (95% CI, 0.63 to 0.92) in the validation set. Although the nomogram predicted probabilities were lower than the actual probabilities, dichotomous outcomes showed only a few false negatives in both sets and high negative predictive values in the training set (88.0%; 95% CI, 0.79 to 0.95) and validation set (77.8%; 95% CI, 0.60 to 0.87), which implies that the cutoff value was still effective under underestimated conditions. These results suggested that the performance of dichotomous outcomes could be generalizable to other populations. The introduction of a cutoff value to the nomogram was advantageous on three counts: to increase clinical convenience and practicality, to allow the integration of surgical resection into the nomogram, and to compensate for the underestimation of actual probabilities.

**Table 4:** **Two way contingency table analysis showing predictive accuracy of the nomogram**

		Training set			Validation set		
		Observed (***N***)			Observed (***N***)		
		Metastasis positive	Metastasis free	Total	Metastasis positive	Metastasis free	Total
Expected (*N*)	Metastasis positive	28	13	41	14	2	16
	Metastasis free	6	44	50	4	14	18
	Total	34	57	91	18	16	34
Accuracy % (95% CI)		79.1 (0.69 to 0.86)			82.4 (0.63 to 0.92)		
Sensitivity % (95% CI)		82.4 (0.69 to 0.92)			77.8 (0.60 to 0.87)		
Specificity % (95% CI)		77.2 (0.69 to 0.83)			87.5 (0.67 to 0.98)		
PPV % (95% CI)		68.3 (0.57 to 0.76)			87.5 (0.67 to 0.98)		
NPV % (95% CI)		88.0 (0.79 to 0.95)			77.8 (0.60 to 0.87)		
PLR (95% CI)		3.61 (2.21 to 5.36)			6.22 (1.83 to 35.63)		
NLR (95% CI)		0.23 (0.10 to 0.456)			0.25 (0.14 to 0.60)		
DOR (95% CI)		15.80 (4.84 to 54.44)			24.5 (3.07 to 261.90)		

*Abbreviations*: *CI* confidential interval, *PPV* positive predictive value, *NPV* negative predictive value, *PLR* positive likelihood ratio, *NLR* negative likelihood ratio, *DOR* diagnostic odds ratio.

## Discussion

To construct a nomogram with better performance, it is more advantageous to use a large training set and many prognostic factors with strong correlations to an event. On the other hand, inclusion of too many predictors compared to size of training set and overly complicated parameters of predictors are likely to result in an overfitted prediction model. Osteosarcoma is a rare disease and only a few well-validated prognostic factors for metastasis have been identified, which is likely to make prediction model overfitted. To overcome this and increase statistical simplicity of the nomogram, we limited the numbers of predictors used to build the nomogram according to the guidelines of Harrell [14]. In addition, we divided the parameters of all predictors into only two prognosis groups, good or poor. Whether the performance of the nomogram is reproducible in other populations is more important than overfitting. We validated the reproducibility of our nomogram in external validation set, which was heterogeneous to the training set with respect to surgeon factor and surgery type (limb salvage or amputation). The validation results suggested that our nomogram could be generalizable to other patient populations, including populations with amputation rather than limb salvage surgery.

It has been a general consensus that the prognosis of osteosarcoma with axial and proximal locations is poorer than that of osteosarcoma with distal locations [5, 12]. However, the prognosis of osteosarcoma with proximal humeral locations is controversial [6, 7]. Because the results of our study were similar to those reported by Meyers et al., osteosarcomas with proximal humeral location were classed as good prognosis group in our nomogram.

Although the effective cutoff range is still uncertain, tumor size has been reported as a definitive prognostic factor in osteosarcoma [20, 21]. Although the cutoff of 8 cm in maximal tumor diameter was not a prognostic factor for metastasis in our study, we integrated tumor size into our nomogram for clinical considerations. We integrated the effect of large tumor size into tumor site by defining large tumors exceeding the isthmus of the affected bone (more than half of the entire length of the affected bone) as the poor prognosis group, as one would expect that such a large tumor would show a poor prognosis. As a result, very large tumors were classified as poor prognosis group despite their primary location.

Tumor invasion of the joints with direct penetration through the articular cartilage are expected to be rare in osteosarcoma because articular cartilage acts as a strong barrier to tumor invasion. However, it has been reported that intracapsular and extrasynovial involvements are common in osteosarcoma [22, 23]. Tumors can extend under the joint capsule and make contact with the peripheral margin of the articular cartilage. In the case of knee joints, tumors can also extend through or around the osseoustendinous junction of the cruciate ligaments. We defined intracapsular extension of the tumor as extension into the intracapsular and extrasynovial structures as well as the penetration through articular cartilage by tumors. The use of MRI to identify intracapsular extension is limited because its high sensitivity makes it difficult to distinguish peritumorous inflammatory changes and edema from the tumor itself, which results in false-positives [24]. To overcome this, we confirmed intracapsular extension by MRI and gross pathology.

Complete surgical resection of tumor has also been regarded as a definitive prognostic factor of osteosarcoma. However, it may be questionable to assign a cutoff value for incomplete surgical resection because the strength of the association between incomplete surgical resection and metastasis has not been proven quantitatively. Inadequate surgical margin (marginal and intralesional margin) had a relative risk of approximately 1.4 for event-free survival or metastasis when compared to adequate surgical margin (radical and wide margin) [25, 26]. On the basis of these data, the importance of incomplete surgical resection is likely to be highly underestimated if it is not taken into consideration that residual tumor is not retained in all marginal margins. In fact, osteosarcoma with incomplete surgical resection to retain macroscopic residual tumor showed a 5-year survival rate of only 15% and a relative risk for overall survival of 3.60 in the multivariate analysis when compared to complete surgical resection, which was higher than the relative risks of metastasis positive at presentation [12]. We obtained similar results in our study, although all the incomplete surgical resection cases in our study were microscopically margin positive.

As survival rates of osteosarcoma increase, the prognoses of individual patients become of greater interest. AJCC and Enneking staging system have been used to classify prognostic groups after initial assessments. However, high grade osteosarcoma shows a clinical course so heterogeneous during treatment that the prognoses of individual osteosarcomas may widely vary, even if their initial stages, such as AJCC classification or Enneking system, are the same. Therefore, a nomogram may be useful in the management of osteosarcoma to realize personalized prognoses. Survival rates of osteosarcoma with metastasis are approximately 20% and early detection and aggressive metastasectomy should be considered to increase survival rates of patients with metastasis [18]. Accordingly, distinguishing patients at high risk for metastasis according to the nomogram and swift management of metastatic lesions may comtribute to improvement in survival rates forosteosarcoma.

Our nomogram had several limitations. First, our training set was relatively small and had a deviated composition of Asian. In addition, our validation set was quite small and showed a higher proportions of patients with metastasis than those of natural populations, as considerable number of patients with CDF and NED status at less than 5 years were excluded from cohort 2 due to a short follow-up period. The generalizability of our nomogram should be validated in larger populations with a natural proportion of patients with metastasis. Second, our nomogram underestimated actual probabilities presented as percentage. To avoid inaccurate predictions, dichotomous outcomes should be considered because it was less affected by underestimation. Third, the predictors used to construct our nomogram were confined to clinical factors and could not include molecular markers. Fourth, our nomogram cannot predict the time when metastasis occurs because it was based on logistic regression and not Cox regression. A positive dichotomous decision for metastasis without any indication of time of occurrence may be unnerving to patients and doctors.

## Conclusions

We have developed a new postoperative nomogram with high performance and generalizability to predict the probability of metastasis in Enneking stage IIB extremity osteosarcoma. Development of this nomogram will contribute greatly to individualized risk assessments for metastasis in osteosarcoma.

## Abbreviations

AJCC: American Joint Committee on Cancer
ALP: Alkaline phosphatase
LDH: Lactate dehydrogenase
AUC: Area under receiver operating characteristic curve
LSS: Limb salvage surgery
CDF: Continuously disease free
DOD: Died of disease
NED: No evidence of disease
AWD: Alive with metastatic disease
DOC: Died of other cause
NA: Not available
PPV: Positive predictive value
NPV: Negative predictive value
PLR: Positive likelihood ratio
NLR: Negative likelihood ratio
DOR: Diagnostic odds ratio.
